# Sufferers Who Long to Die

**Published:** 1895-09

**Authors:** 


### Sufferers Who Long to Die.

The old subject of euthanasia, has been raised again in the pages of a contemporary, wherein it is discussed at some length, and with quotatations alleged to be from the conversation of ''eminent'' physicians who are said to have spoken in favor of poisoning any unfortunate patient who may desire release from his sufferings. One medical man, anonymous we need not say, speaks (according to the interviewer) quite complacently of his share in a little murder of this kind, though he begs the reporter not to say too much about it; and another relates a con_

spiracy of the sort with a wealthy and harassed patient, and admits that he was not indisposed to assist in the deed, but held his hand in fear the parent might peach when his gratitude or his temper wore out. Well do we remember an evening at a private medical society when a medical friend produced a full-time fetus, sufficiently hideous, no doubt, which he had—well, not attempted to bring to life. When the monster had the temerity to show signs of respiration, our friend was so aghast that he dropped it into the wash-hand basin. The naive innocence of his story disarmed his auditors. We do not intend seriously to discuss this old problem over again, which would lead us far, far into ethics, far into religion, but we may remind our readers of the old saying, "Hard cases make bad law," and the number of such cases is exceeding few. People may debate the matter in the abstract, but physicians in the largest practice will find it difficult to adduce an instant in which the precise psychological moment presented itself to a sane patient and sane friends. Of the insane we are not speaking. To suggest that physicians, in the secrecy of the bed-chamber, are to hold themselves ready to practice Thuggee on their patients, either on the patient's own suggestion or to please distraught or designing bystanders, would be absurd if it were not so horrible. One thorough-going euthanasiast we have met who had sense of the decent and fitting. His proposition was that after a due inquiry before a justice, the victim should be executed by the bishop of the diocese. Episcopal journals please copy.—*British Medical Journal.*

